# The influence of different intraocular pressure on lamina cribrosa parameters in glaucoma and the relation clinical implication

**DOI:** 10.1038/s41598-021-87844-1

**Published:** 2021-05-07

**Authors:** Jian Wu, Yifan Du, Jiaying Li, Xiaowei Fan, Caixia Lin, Ningli Wang

**Affiliations:** 1 Beijing Institute of Ophthalmology, Beijing Tongren Eye Center, Beijing Tongren Hospital, Capital Medical University, Beijing, 100730 People’s Republic of China; 2 Beijing Key Laboratory of Ophthalmology and Visual Sciences, No. 1 Dong Jiao Min Xiang Street, Dongcheng District, Beijing, 100730 People’s Republic of China; 3 Department of Ophthalmology, Stanford University School of Medicine, Palo Alto, 94043 CA USA

**Keywords:** Eye diseases, Risk factors

## Abstract

Elevated intraocular pressure (IOP) is one of the main risk factors for glaucoma, and pathological changes in the lamina cribrosa (LC) may play a leading role. This study aimed to explore the influence of different IOP on LC parameters and the correlation between parameters and glaucoma severity. A total of 91 eyes were examined by swept-source OCT and divided into IOP ≥ 30 mmHg (group A), 21 mmHg ≤ IOP < 30 mmHg (group B), and normal IOP (control, group C). Clinical parameters and all LC parameters such as cup depth (CD), lamina cribrosa depth (LCD), prelaminar tissue thickness (PTT) and LC curvature index (LCCI) were used for statistical analysis. The bulk of parameters were greater in group A than in the other groups (group B, P < 0.05; group C, P < 0.001). PTT and Bruch’s membrane opening minimum rim width (BMO-MRW) were thinner in group A than in group C (P < 0.01). In univariate and multivariable linear regression analysis, visual field (VF), mean retinal nerve fiber layer (RNFL) thickness, CD, LCD, PLCSD, PTT, LCCI, aLCCI, and BMO-MRW were significantly correlated with IOP changes (P < 0.05). Pearson test showed that LCD and LCCI were correlated with mean retinal nerve fiber layer (RNFL) thickness (LCD, r = − 0.420, P = 0.002; LCCI, r = − 0.449, P < 0.001) and BMO-MRW (LCD, r = − 0.245, P = 0.019; LCCI, r = − 0.345, P < 0.001). Therefore, different levels of IOP have a remarkable effect on clinical symptoms (VF, BCVA) and LC parameters, between which there may be a linear relationship. LCCI may exhibit a more significant correlation with RNFL thickness and BMO-MRW, which may further suggest that LCCI shows a better correlation with clinical symptoms under the influence of long-term high IOP.

## Introduction

Glaucoma is the leading cause of vision loss and irreversible blindness worldwide^1^. POAG, as one of the main types of glaucoma^2^, is characterized by atrophy and depression of the optic papilla, visual field (VF) defects, and visual loss, manifesting as the loss of retinal ganglion cell and axon functions^3,4^. Moreover, with its insidious onset acting as an inhibiting factor, identifying the early stages of POAG is extremely difficult. The pathological increase in intraocular pressure (IOP) is the major risk factor for POAG^5^. Therefore, it has become increasingly important to discover an effective examination method, as well as a more accurate diagnostic standard for the early detection of POAG.

The early pathological changes of POAG were first defined based on deformation and damage of the lamina cribrosa (LC), which can be mainly attributed to two aspects: mechanical or ischemic. LC compression and backward bowing can incur kinking and pinching of axons directly in the laminar pores, which leads to blockage or aggravation of the blockage of axonal flow^6–8^. The blood flow disorder elicited by a deformed LC can reduce nutrition and the oxygen supply and can accelerate retinal ganglion cell (RGC) apoptosis^7,9^. The mechanism of LC deformation and ischemia may be associated with the change in POAG. However, regardless of the theory, post deformation of the LC is the basis of POAG^10–13^.

Examination of the postdeformation LC has always posed a major problem in ophthalmology. In early ophthalmic examinations, an important method to diagnose POAG is enlargement of the cup/disk ratio (CDR) and retinal nerve fiber layer (RNFL) defects in the corresponding region^14^. With the advent of OCT, which can scan the deep structure of the optic nerve head (ONH), observation of the LC structure has a new trend of development. Moreover, an emerging hot spot is to evaluate the change in the LC by OCT scans for ONH^15^. In recent years, with the use of swept-source (SS)-OCT and enhanced depth imaging (EDI)-OCT, which have better tissue penetration, it has become possible to evaluate LC parameters more clearly and accurately^6,16,17^.

With the development and update of OCT, a growing number of LC parameters have been mined to evaluate the progression of POAG. A potential indicator of LC morphology is the LC depth (LCD) since it is correlated with the postdeformation magnitude of LC. Furthermore, compared with that in the control group, the LCD in glaucoma patients was significantly increased in the previous study^18^. At the same time, the prelaminar tissue thickness (PTT) of both the affected eyes and the contralateral healthy eyes of glaucoma patients was thinner^19^. The LC curvature index (LCCI) may be another useful parameter for LC morphological changes. Compared with healthy control eyes, POAG eyes have a larger LCCI, and the LCCI has a better diagnostic performance for glaucoma than LCD^20^. Moreover, as an index excluding the influence of Bruch membrane opening (BMO) width, adjusted LCCI (aLCCI) has also been proven to be a stable parameter related to glaucoma^21^. Additionally, cup depth (CD), lamina cribrosa thickness (LCT), Bruch's membrane opening minimum rim width (BMO-MRW) and other LC parameters have also been confirmed to be correlated with glaucoma^22–25^. Furthermore, IOP, as a major influencing factor of POAG, has been found to be closely associated with relevant LC parameters^26,27^. An increase in IOP can affect LCD and LCCI. In an experimental study of induced IOP rise, Jiang et al. found a significant widening and deepening of the optic cup, as well as thinning of the LC^28^. In POAG patients, LCD is positively correlated with IOP. In addition, several studies have demonstrated that the LC moves forward and the prelaminar tissue is thickened in POAG patients^29,30^ when high IOP is relieved. However, the mechanism and whether the level of IOP affects the LC remain unknown.

IOP is an important factor in the pathogenesis of POAG; thus, it is critical to explore the influence of the IOP level on LC parameters and the mechanism of IOP in the LC for early detection of POAG progression. The purpose of this study is to use SS-OCT with better tissue penetration to explore the influence of different IOP levels on LC parameters, how IOP affects LC changes, and in turn, to further inference the relationship between LC parameters and disease severity under the influence of different IOPs. Finally, we expect to provide directions for further studies to find effective methods for the early diagnosis of POAG and to prevent its progression.

## Methods

### Study participants and procedure

This prospective observational study was approved by the Beijing Tongren Hospital Institutional Review Board and adhered to the tenets of the Declaration of Helsinki. Written informed consent was obtained from all patients. POAG patients who were admitted to Beijing Tongren Hospital and volunteers with normal IOP who did not have POAG were included as subjects in this study. To qualify for the study, POAG patients met the following criteria: glaucomatous optic disc changes, such as diffuse or localized notching, retinal nerve fiber layer (RNFL) defects in stereo disc photography, glaucomatous VF defects corresponding to structural changes, and open angle confirmed by gonioscopic examination, were treated with medication for lower IOP (Beta Blockers, Adrenergic Agonists, Carbonic Anhydrase Inhibitors, etc.) but IOP maintained with a constant high level. Normal IOP subjects without POAG were defined as individuals who regularly had gone to the Beijing Tongren Hospital for eye examination (e.g., dry eyes, cataract) and did not present RNFL defects in stereo disc photography, red-free RNFL photography, and standard automatic VF examination, with a normal IOP after multiple examinations. In view of the positive correlation between binocular subjects in statistical analysis, we stipulated that binocular subjects were not included in the same group in order to exclude this effect from the final analysis. That is to say, whether the binocular subjects had either one eye being completely normal and the other eye having POAG with high IOP, or both eyes had POAG, the difference of IOP and disease progression were large.

All selected subjects underwent a comprehensive ophthalmic evaluation: visual acuity assessment, slit-lamp biomicroscopy, gonioscopy, Goldmann applanation tonometry (Haag Streit, Koniz, Switzerland), dilated fundus examination, digital color stereo disc photography (vx-10; Kowa Optimed, Tokyo, Japan), central corneal thickness measurement (Orbscan 73 II; Bausch & Lomb Surgical, Rochester, NY), axial length (AL) measurement (Axis II PR; Quantel Medical, Inc., Bozeman, MT), and central 30–2 threshold test of Humphrey visual field (HFA II; Humphrey Instruments, Inc., Dublin, CA). Moreover, experienced ophthalmologists performed SS-OCT when the pupil was completely dilated.

All subjects were followed up every three months. IOP was the follow-up indicator in this study. Before the subjects were included, it was ensured that they had available IOP values from the previous three follow-up visits (once every three months, with a total follow-up time of approximately half a year to one year). During each visit, IOP was evaluated through Goldmann applanation tonometry. All examinations (excluding IOP) in this study were performed at each patient’s latest follow-up.

The inclusion criteria of subjects were as follows: aged 18–75 years, best-corrected visual acuity (BCVA) ≥ 20/40, and IOP without fluctuation in a large range (IOP fluctuation ≥ 20 mmHg) between each follow-up visit. Subjects did not receive any glaucoma surgical interventions in the course of the disease before being included in this study. The exclusion criteria included other types of glaucoma, except POAG, optic disc drusen, anterior ischemic optic neuropathy, optic disc disease, retinal disease (such as macular degeneration, diabetic retinopathy, retinal arteriovenous occlusion, etc.), and other ophthalmic diseases similar to glaucoma, such as optic neuropathy. Over the last three months, ophthalmic operation was carried out, and patients lacking high quality OCT images and stereo disc photography were excluded. At the same time, POAG patients met the condition of high IOP in all follow-ups. Normal control subjects met the conditions of normal IOP, and no glaucoma changes were found in ophthalmic examinations.

### Measurement and grouping of IOP

Calibrated Goldmann applanation tonometry was used to measure IOP in this study. The IOP was measured simultaneously with the same equipment during each follow-up. The patients maintained the same posture for adequately determining the average value of IOP. The mean IOP was calculated based on the average value of IOP at all follow-up visits. The highest IOP was defined as the top IOP value measured during the entire follow-up period, instantaneous IOP referred to the IOP measurement value before the participants underwent SS-OCT examination, and the mean IOP was the index of final grouping. Therefore, all subjects were divided into three groups based on the mean IOP in the previous year: IOP ≥ 30 mmHg (group A, n = 30), 21 mmHg ≤ IOP < 30 mmHg (group B, n = 33), and normal IOP (control, group C, n = 28). In the following study, IOP was divided into six groups with intervals of 5 mmHg, and the average value of each group is shown in a line graph to roughly explore the development of LC parameters at different IOP levels.

### Swept-source OCT imaging of optic disc

All eyes were scanned using a DRI OCT-1 Atlantis 3D SS-OCT device (Topcon Medical Systems, Oakland, NJ). The baseline morphological features of the LC were evaluated from 12 SS-OCT radial line B-scans centered on the optic disc, with each scan at a half clock-hour meridian. All final LC parameters were obtained by averaging the results of one o’clock (transverse scan) and seven o’clock (longitudinal scan); thereafter, the comprehensive results were applied for further analysis. All scanning results were rechecked prior to analysis to determine whether the peripheral LC display was poor due to serious vascular shadows or local LC defects. Furthermore, OCT scanning results with poor overall image quality were excluded. Eyes with poor peripheral LC on three scans were excluded from the study. The remaining energy was computed for all A-Scans of a given image. The maximum remaining energy (which corresponds to the A-Scan exhibiting the least attenuation) then was then computed as,

$$E_{i}^{\max } = \mathop {\max }\limits_{j} [E_{i,j} ]$$

where i is the OCT pixel index for a given A-Scan j. This remaining energy profile, indicating the maximum penetration profile, is then used for thresholding, to determine the depth at which the compensation factor is kept constant, which is then used for all AScans. This approach avoids stopping the compensation prematurely in regions of strong attenuation, as it determines the limit from the A-Scans with smaller attenuation. Additionally, we obtained prior permission from the algorithm developer^31–33^.

### Measurement of LC parameters

**Figure 1:**
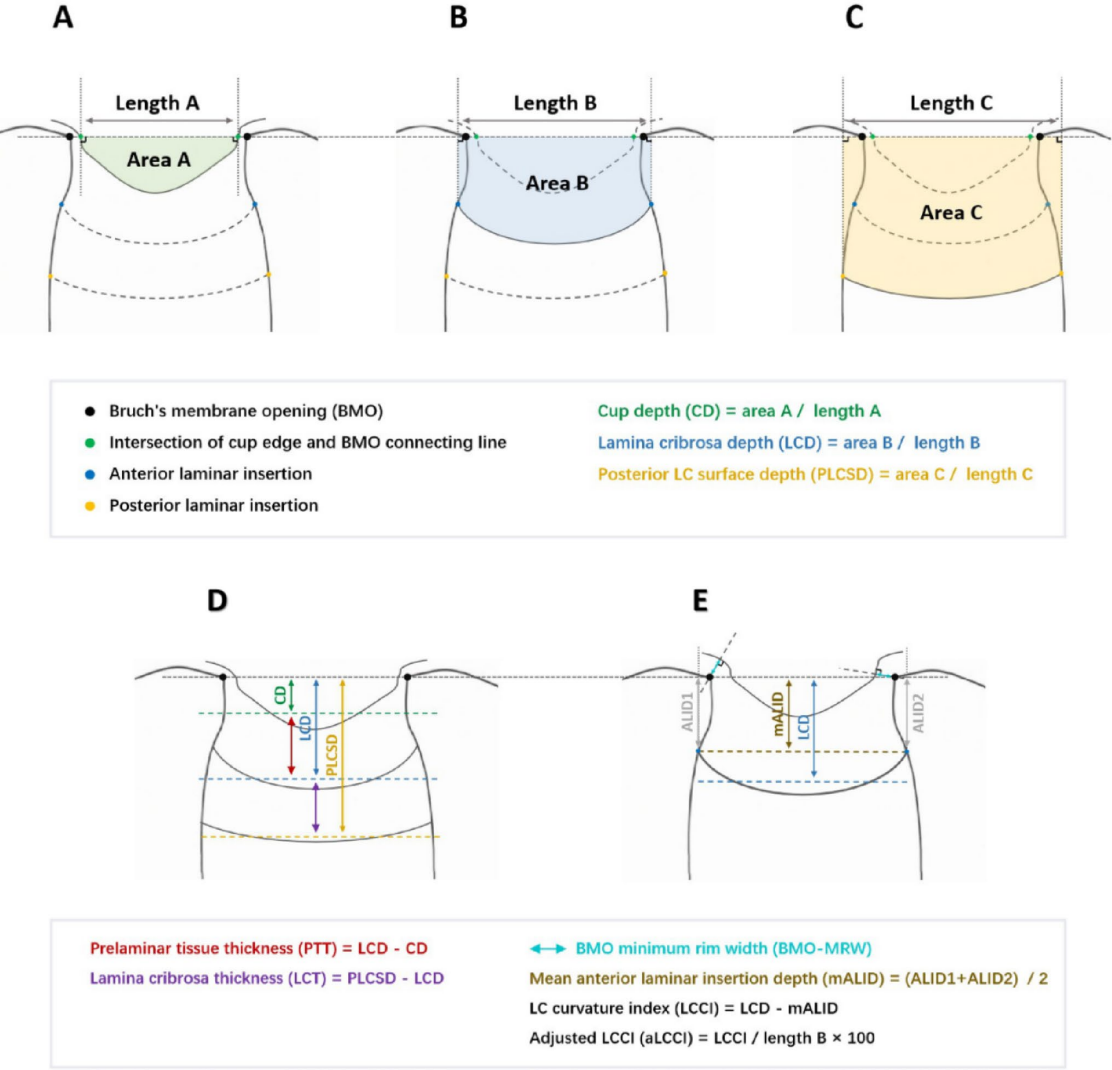
Examples of measurement methods of various lamina cribrosa related parameters. The three figures above showed the measurement of the three depth indicators (CD, LCD, PLCSD). Their calculation methods are similar. They all approximate the depth index by making two vertical lines perpendicular to the BMO line, calculating the middle area (colored area in the figure) and dividing by the length of the bottom edge. The two figures below showed the measurement of other screening parameters. The thickness index is obtained by subtracting the depth index calculated at the front edge. BMO-MRW refers to the shortest vertical distance between BMO and ILM, ALID refers to the vertical distance from lamina cribrosa edge to BMO line, and LCCI refers to the difference between average ALID and LCD on both sides. aLCCI is defined as LCCI divided by the length of bottom edge, which can exclude the mixed effect of BMO opening width on LCCI.

All measurements were performed using ImageJ software (National Institutes of Health, Bethesda, MD). The methods of the measurements are shown in Fig. 1. All parameters were measured vertically based on the reference line, connecting the BMOs. CD is defined as the average vertical distance from the cup surface to the reference line. Considering that the ONH is most often described by estimating the cup-to-disc ratio, the estimation of CDR is one of the main clinical indicators for differentiation between normal and glaucomatous ONH and for assessing glaucomatous changes over time. LCD is the average vertical distance from the front surface of the LC to the reference line; posterior lamina cribrosa surface depth (PLCSD) is defined as the average vertical distance from the back surface of the LC to the reference line. For the average measurement to reflect the overall scenario as much as possible, the method of the overall region divided by the bottom-edge length was adopted in this study as the measurement result of the above parameters (Fig. 1A–C)^34^.

In this study PTT was defined as the distance from the cup surface to the front surface of the LC, and LCT was defined as the distance between the front and back surfaces of the LC. These two parameters were obtained by directly subtracting relevant parameters (Fig. 1D). The anterior laminar insertion depth (ALID) was described as the vertical distance between the anterior laminar insertion and the reference line. In this study, the average value of ALID on both sides of the LC plane was considered as the mean value(mALID). Additionally, a modified LCCI parameter, i.e., adjusted LCCI (aLCCI), was also introduced by referring to relevant articles that considered the steepness of the LC curve^21^. Even with identical LCCIs, the smaller the lengths of A, B, and C, the steeper the LC curve becomes (Fig. 1A–C,E). In 91 randomly selected eyes, all parameters were independently measured by two experienced ophthalmologists (JW and YFD), who were unaware of the clinical information of each patient, to exclude the tendency of measurement results.

### Data analysis

SPSS software (version 25.0; SPSS, Chicago, IL, USA) was used for statistical analysis. Data for each group were compared by an independent t-test. The independent variables included age, sex, eye type, CCT, BCVA, AL, mean IOP, maximum IOP, instantaneous IOP, cup disk ratio (CDR), cup volume (CV), and mean RNFL thickness. In addition, univariate linear regression analysis and multivariable linear regression analysis were used to analyze the relationship between the mean IOP and the factors. When P < 0.05, significant variables were included in the multivariate model. Afterwards, Pearson correlation analysis was utilized to assess the strength of the relationship between the representative parameters of LC (LCD and LCCI) and the RNFL thickness (mean RNFL thickness and mean BMO-MRW) of glaucoma under different IOP boundaries^35^. P < 0.05 was considered statistically significant.

## Results

### Demographic and clinical characteristics of participants

A total of 132 eyes were included for examination at the beginning of this study, of which 19 were excluded due to lack of relevant clinical data (CCT, AL, stereo disc photography, etc.). Ten eyes were excluded due to failure to meet the number of IOP measurements required in this study. In the final process of parameter measurement, another 12 eyes were excluded due to image quality deviation. Following the screening, 182 OCT images of 91 eyes were analyzed.

The final sample capacity of the study consisted of 91 eyes from 57 participants whose average age was 44.6 ± 15.6 years. There were 37 males (64.9%) and 20 females (35.1%). The average of the patients suffered from POAG (mean duration of POAG, refer to the time from first visit to the hospital to involve in this study) were 1.2 ± 0.3 years, and most of the patients had the disease for more than one year. Among the 91 eyes, 48 were left eyes (52.7%) and 43 were right eyes (47.3%). The duration of glaucoma in the patients ranged from 9 months to 22 years The mean IOP of the samples was 25.9 ± 8.8 mmHg (range 12–55 mmHg); the highest IOP was 31.5 ± 12.0 mmHg (range 14–70 mmHg), while the instantaneous was 22.5 ± 10.4 mmHg (range 9–60 mmHg). The clinical characteristics of the participants are summarized in Table 1.

**Table 1:** Clinical characteristics of study participants (n = 91).

Characteristics	Values
Age (years)	44.6 ± 15.6
Gender (male/female)	59/32
Eye (OD/OS)	43/48
Mean duration of POAG (y)	1.5 ± 0.7
BCVA	0.5 ± 0.4
Axial length (mm)	24.3 ± 2.6
VF MD (dB)	− 14.5 ± 9.6
CCT (μm)	500.0 ± 32.9
IOP (mmHg)	
Instant IOP (mmHg)	22.5 ± 10.4
Maximum IOP (mmHg)	31.5 ± 12.0
Mean IOP (mmHg)	25.9 ± 8.8
CDR	
Mean CDR	0.7 ± 0.2
Mean scan surface CDR	0.7 ± 0.2
CV (mm^3^)	0.6 ± 0.4
Mean RNFL thickness (mm)	64.0 ± 26.3

BCVA = best-corrected visual acuity; CCT = central corneal thickness; CDR = cup-disk ratio; CV = cup volume; IOP = intraocular pressure; MD = mean deviation; POAG = Primary open-angle glaucoma; RNFL = retinal nerve fiber layer; VF = visual field.

Values are mean ± standard deviation.

### Comparison of basic data among three groups

There were no differences in terms of age, sex, eye type, central corneal thickness, or AL among the three groups (P > 0.05). The BCVA of group A (0.2 ± 0.2) was significantly lower than that of group B (0.5 ± 0.4, P = 0.004) and group C (0.6 ± 0.3, P < 0.001), however, there was no significant difference between groups B and C (P = 0.190). The VF mean deviation (MD) of group A (− 21.3 ± 7.6) was significantly lower than that of group B (− 15.6 ± 7.8, P = 0.008) and group C (− 5.4 ± 5.5, P < 0.001). In the meantime, there was also a significant difference between groups B and C (P < 0.001). In addition, the highest and instantaneous IOPs in group A (44.0 ± 8.4 mmHg, 29.2 ± 11.1 mmHg) were higher than those in group B (29.3 ± 8.0 mmHg, P < 0.001; 20.7 ± 9.7 mmHg, P = 0.002) and group C (20.8 ± 5.4 mmHg, P < 0.001; 17.4 ± 5.7 mmHg, P < 0.001). Meanwhile, the highest IOP in group B was significantly higher than that in group C (P < 0.001), but there was no significant difference in instantaneous IOP between groups B and C (P = 0.121). The mean CDR, mean scan surface CDR, and CV of group A (0.8 ± 0.1, 0.8 ± 0.1, 0.8 ± 0.3mm^3^, respectively) were significantly higher than those of group C (0.6 ± 0.2, 0.6 ± 0.2, 0.4 ± 0.3mm^3^, respectively, P < 0.001), however, only the CV was markedly higher than that of group B (0.6 ± 0.4mm^3^, P = 0.008). Moreover, the mean CDR and mean scan surface CDR of group B (0.7 ± 0.2, P = 0.013, 0.7 ± 0.2, respectively, P = 0.004) was significantly higher than that in group C. Although the mean RNFL thickness of group A (42.8 ± 12.7 mm) was obviously smaller than that of group B (69.2 ± 19.9 mm, P < 0.001) and group C (80.6 ± 29.2 mm, P < 0.001), but there was no difference between groups B and C (P = 0.075). The comparison results among the three groups of parameters are illustrated in Table 2.

**Table 2:** Comparison of baseline parameters of study participants with IOP ≥ 30 mmHg (Group A), 21 mmHg ≤ IOP < 30 mmHg (Group B), and normal IOP (Group C).

	Group A	Group B	Group C	*P* Value_A-B_	*P* Value_A-C_	*P* Value_B-C_
No. of eyes	30	33	28			
Age (years)	45.2 ± 18.2	41.1 ± 13.1	48.1 ± 14.9	0.300	0.509	0.504
Male sex (%)	66.7	72.7	53.6	0.608	0.317	0.125
OD (%)	53.3	42.4	46.4	0.395	0.607	0.759
BCVA	0.2 ± 0.2	0.5 ± 0.4	0.6 ± 0.3	**0.004**	**< 0.001**	0.190
Axial length (mm)	24.5 ± 2.1	23.9 ± 3.1	24.7 ± 2.6	0.359	0.792	0.300
VF MD (dB)	− 21.3 ± 7.6	− 15.6 ± 7.8	− 5.4 ± 5.5	**0.008**	**< 0.001**	**< 0.001**
CCT (μm)	502.3 ± 38.4	500.7 ± 33.9	496.8 ± 26.0	0.865	0.541	0.630
Instant IOP (mmHg)	29.2 ± 11.1	20.7 ± 9.7	17.4 ± 5.7	**0.002**	**< 0.001**	0.121
Maximum IOP (mmHg)	44.0 ± 8.4	29.3 ± 8.0	20.8 ± 5.4	**< 0.001**	**< 0.001**	**< 0.001**
Mean CDR	0.8 ± 0.1	0.7 ± 0.2	0.6 ± 0.2	0.446	**< 0.001**	**0.013**
Mean scan surface CDR	0.8 ± 0.1	0.7 ± 0.2	0.6 ± 0.2	0.257	**< 0.001**	**0.004**
CV (mm3)	0.8 ± 0.3	0.6 ± 0.4	0.4 ± 0.3	**0.008**	**< 0.001**	0.066
Mean RNFL thickness (mm)	42.8 ± 12.7	69.2 ± 19.9	80.6 ± 29.2	**< 0.001**	**< 0.001**	0.075
BMO (μm)	1722.3 ± 213.8	1736.5 ± 408.9	1733.3 ± 203.1	0.865	0.841	0.970
Cup depth (μm)	547.8 ± 202.6	342.8 ± 221.5	161.6 ± 166.0	**< 0.001**	**< 0.001**	**< 0.001**
LCD (μm)	655.3 ± 173.9	513.9 ± 154.8	404.5 ± 102.9	**0.001**	**< 0.001**	**0.002**
PLCSD (μm)	876.8 ± 160.4	739.7 ± 152.1	648.2 ± 124.4	**< 0.001**	**< 0.001**	**0.014**
PTT (μm)	107.5 ± 42.6	171.1 ± 131.2	242.9 ± 142.3	**0.014**	**< 0.001**	**0.045**
LCT (μm)	221.4 ± 69.8	225.8 ± 46.6	243.7 ± 47.3	0.770	0.163	0.142
LCCI (μm)	76.2 ± 28.0	58.0 ± 33.8	35.8 ± 29.2	**0.024**	** < 0.001**	**0.009**
aLCCI (μm)	6.7 ± 2.4	4.5 ± 2.0	3.4 ± 2.4	**< 0.001**	**< 0.001**	**0.043**
BMO-MRW	129.6 ± 38.0	154.6 ± 67.3	195.1 ± 99.6	0.078	**0.001**	0.064

aLCCI = adjusted lamina cribrosa curvature index; BCVA = best-corrected visual acuity; BMO = Bruch’s membrane opening; BMO-MRW = Bruch’s membrane opening minimum rim width; CCT = central corneal thickness; CDR = cup-disk ratio; CV = cup volume; IOP = intraocular pressure; LCCI = lamina cribrosa curvature index; LCD = lamina cribrosa depth; LCT = lamina cribrosa thickness; MD = mean deviation; PLCSD = posterior lamina cribrosa surface depth; PTT = prelaminar tissue thickness; RNFL = retinal nerve fiber layer; VF = visual field.

All values < 0.05 at t-test are presented in boldface.

### Comparison of LC parameters among three groups

**Figure 2:**
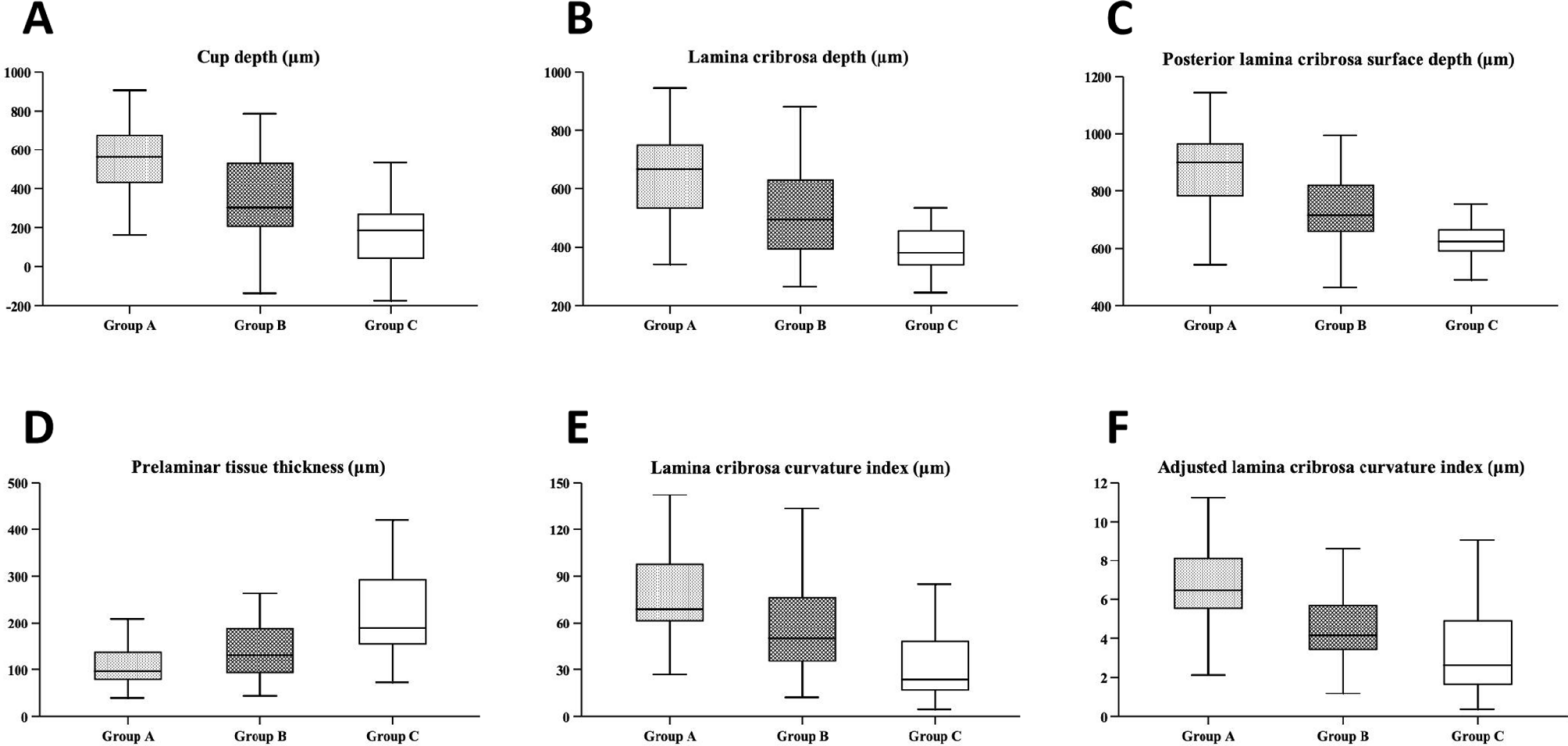
Box diagram of related parameters of lamina cribrosa between three groups (**A**–**F**) showed the overall distribution of CD, LCD, PLCSD, PTT, LCCI and aLCCI among the three groups, respectively. From the comprehensive depth index (**A**–**C**), group A (IOP ≥ 30 mmHg) was significantly deeper than Group C (Normal control), and followed by group B (21 mmHg ≤ IOP < 30 mmHg), and the same performance was shown in curvature index (**E**,**F**). In the PTT (**D**), the performance of the three groups was opposite to that of the anterior index, that is, PTT with group A (IOP ≥ 30 mmHg) was significantly thinner.

In a comparison among the three groups, CD, LCD, and PLCSD in group A (547.8 ± 202.6 μm, 655.3 ± 173.9 μm, 876.8 ± 160.4 μm, respectively) was significantly deeper than those in group B (342.8 ± 221.5 μm, P < 0.001; 513.9 ± 154.8 μm, P = 0.001; 739.7 ± 152.1 μm, P < 0.001) and group C (161.6 ± 166.0 μm, 404.5 ± 102.9 μm; 648.2 ± 124.4 μm, P < 0.001). Simultaneously, the three parameters of group B were significantly deeper compared than those of group C (CD, P < 0.001; LCD, P = 0.00; PLCSD, P = 0.014). The PTT in group A (107.5 ± 42.6 μm) was significantly thinner than that in group B (171.1 ± 131.2 μm, P = 0.014) and group C (242.9 ± 142.3 μm, P < 0.001). In addition, PTT in group B was significantly thinner than that in group C (P = 0.045). However, there was no significant difference in LCT among the three groups. In terms of LC curvature, the LCCI, and aLCCI of group A (76.2 ± 28.0 μm and 6.7 ± 2.4 μm, respectively) were significantly higher than those of group B (58.0 ± 33.8 μm, P = 0.024; 4.5 ± 2.0 μm, P < 0.001) and group C (35.8 ± 29.2 μm, P < 0.001; 3.4 ± 2.4 μm, P < 0.001). Meanwhile, these two parameters of group B were also significantly higher than those of group C (LCCI, P = 0.009; aLCCI, P = 0.043). For the BMO-MRW, only group A was significantly thinner than group C (P = 0.001). The comparison of LC parameters among the three groups is shown in Fig. 2 and Table 2.

### Linear regression analysis

**Figure 3:**
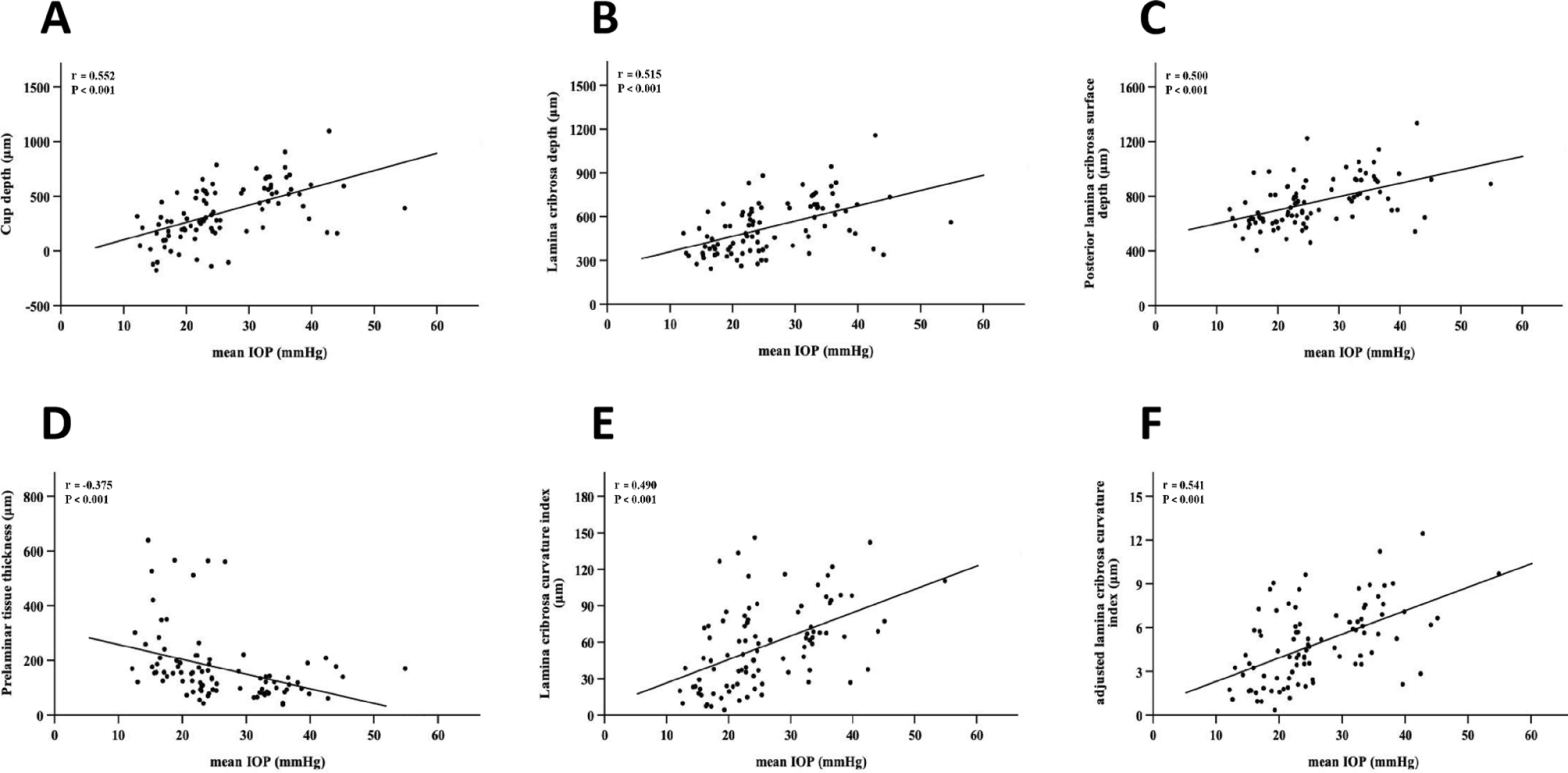
Linear regression analysis between different IOP levels and related parameters of lamina cribrosa. (**A**–**F**) showed the linear correlation between different IOP levels and CD, LCD, PLCSD, PTT, LCCI and aLCCI, respectively. The results show that there is a significant linear relationship between different IOP levels and these six parameters (P < 0.001), and the IOP level is positively correlated with the changes of CD, LCD, PLCSD, LCCI and aLCCI, but negatively correlated with PTT.

In the univariate regression analysis model, IOP showed a significant linear correlation with BCVA (P < 0.001), VF MD (P < 0.001), mean scan surface CDR (P = 0.005), mean CDR (P = 0.024), CV (P < 0.001), mean RNFL thickness (P < 0.001), cup depth (P < 0.001), LCD (P < 0.001), PLCSD (P < 0.001), PTT (P < 0.001), LCCI (P < 0.001), aLCCI (P < 0.001), and BMO-MRW (P = 0.001). Thereafter, the relevant factors were further incorporated into the multivariate regression analysis model. It was indicated in results that VF MD (P < 0.001), the mean RNFL thickness (P = 0.006), CD (P = 0.001), LCD (P = 0.022), PLCSD (P = 0.013), PTT (P = 0.036), LCCI (P = 0.014), aLCCI (P = 0.002), and BMO-MRW (P = 0.004) were significantly correlated with IOP changes (Table 3, Fig. 3).

**Table 3:** Linear regression relationship between IOP and factors.

Variable	Univariate analysis		Multivariate analysis	
	β	*P* value	β	*P* value
Age (years)	− 0.034	0.747		
Gender (male/female)	− 0.048	0.651		
Eye (OD/OS)	0.016	0.879		
BCVA	− 0.482	**< 0.001**	− 0.340	0.168
Axial length (mm)	− 0.029	0.793		
VF MD (dB)	− 0.589	**< 0.001**	− 0.483	**< 0.001**
CCT (μm)	0.103	0.344		
Mean CDR	0.237	**0.024**	− 0.062	0.615
Mean scan surface CDR	0.290	**0.005**	0.105	0.320
CV (mm^y^)	0.402	**< 0.001**	0.286	0.162
Mean RNFL thickness (mm)	− 0.633	**< 0.001**	− 0.237	**0.006**
BMO (μm)	− 0.007	0.947		
Cup depth (μm)	0.552	**< 0.001**	0.562	**0.001**
LCD (μm)	0.515	**< 0.001**	0.471	**0.022**
PLCSD (μm)	0.500	**< 0.001**	0.504	**0.013**
PTT (μm)	− 0.375	**< 0.001**	− 0.438	**0.036**
LCT (μm)	− 0.096	0.363		
LCCI (μm)	0.490	**< 0.001**	0.595	**0.014**
aLCCI (μm)	0.541	**< 0.001**	0.780	**0.002**
Mean BMO-MRW (μm)	− 0.340	**0.001**	− 0.483	**0.004**

aLCCI = adjusted lamina cribrosa curvature index; BCVA = best-corrected visual acuity; BMO = Bruch’s membrane opening; BMO-MRW = Bruch’s membrane opening minimum rim width; CCT = central corneal thickness; CDR = cup-disk ratio; CV = cup volume; IOP = intraocular pressure; LCCI = lamina cribrosa curvature index; LCD = lamina cribrosa depth; LCT = lamina cribrosa thickness; MD = mean deviation; PLCSD = posterior lamina cribrosa surface depth; PTT = prelaminar tissue thickness; RNFL = retinal nerve fiber layer; VF = visual field.

Statistical analysis was performed using the general linear model. Statistically significant values are shown in boldface. Factors with P < 0.1 in the univariate analysis were included from the multivariate analysis. All values < 0.05 at univariate and multivariate analysis are presented in boldface.

### Relationship among LCD, LCCI, and RNFL thickness under different IOPs

Pearson correlation analysis demonstrated that LCD (r = − 0.420, P = 0.002), LCCI (r = − 0.449, P < 0.001), and mean RNFL thickness presented linear correlations at the overall level, and the correlation between LCCI and mean RNFL thickness was slightly greater than that of LCD. After splitting different IOPs (IOP, 21, 25, 30, 35 mmHg), the results showed that even if any IOP level was taken as the boundary, there would be a correlation among LCD, LCCI and the mean RNFL thickness with less than the boundary (P < 0.05) in the group, and the mean RNFL thickness would exhibit a correlation (r = − 0.263, P = 0.037) only when IOP was greater than 21 mmHg that LCD. In the analysis of BMO-MRW, LCD (r = − 0.245, P = 0.019) and LCCI (r = − 0.345, P < 0.001) showed linear correlations with BMO-MRW. In the process of comparing the correlation coefficient, the correlation between LCD and mean RNFL thickness did not show a significant statistical difference when compared to LCCI (u = 0.2372, P = 0.8125). The same results were also presented in BMO-MRW comparison (u = 7275, P = 0.4669). However, in the correlation analysis of RNFL thickness after IOP segmentation, with the improvement of the IOP segmentation line, the correlation between LCCI and mean RNFL thickness gradually presented an obvious trend compared with LCD. Moreover, when IOP ≥ 35 mmHg, the correlation strength between LCCI and mean RNFL thickness was higher than LCD (u = 2.0520, P = 0.0402) (Table 4).

**Table 4:** Pearson correlation of LCD, LCCI and RNFL thickness.

IOP boundary	LCD		LCCI		Comparison of correlation coefficients between LCD and LCCI	
	*r*	P Value	*r*	P Value	*u*	P Value
IOP < 21 mmHg	− 0.405	**0.032**	− 0.527	**0.004**	1.0372	0.2996
IOP ≥ 21 mmHg	− 0.263	**0.037**	− 0.236	0.063	0.1910	0.8485
IOP < 25 mmHg	− 0.283	**0.036**	− 0.362	**0.007**	0.5854	0.5583
IOP ≥ 25 mmHg	− 0.122	0.477	− 0.243	0.153	0.8315	0.4057
IOP < 30 mmHg	− 0.279	**0.029**	− 0.335	**0.008**	0.4103	0.6816
IOP ≥ 30 mmHg	0.078	0.682	− 0.165	0.382	1.6230	0.1046
IOP < 35 mmHg	− 0.368	**0.001**	− 0.348	**0.002**	0.1522	0.8790
IOP ≥ 35 mmHg	0.040	0.889	− 0.263	0.343	**2.0520**	**0.0402**
Total data						
Mean RNFL thickness	− 0.420	**0.002**	− 0.449	**< 0.001**	0.2372	0.8125
BMO-MRW	− 0.245	**0.019**	− 0.345	**< 0.001**	0.7275	0.4669

BMO-MRW = Bruch’s membrane opening minimum rim width; IOP = intraocular pressure; LCCI = lamina cribrosa curvature index; LCD = lamina cribrosa depth; RNFL = retinal nerve fiber layer.

All values < 0.05 at Pearson correlation analysis are presented in boldface.

The *u* value more than 1.96 (P < 0.05) was considered to be statistically significant.

### Representative cases

**Figure 4:**
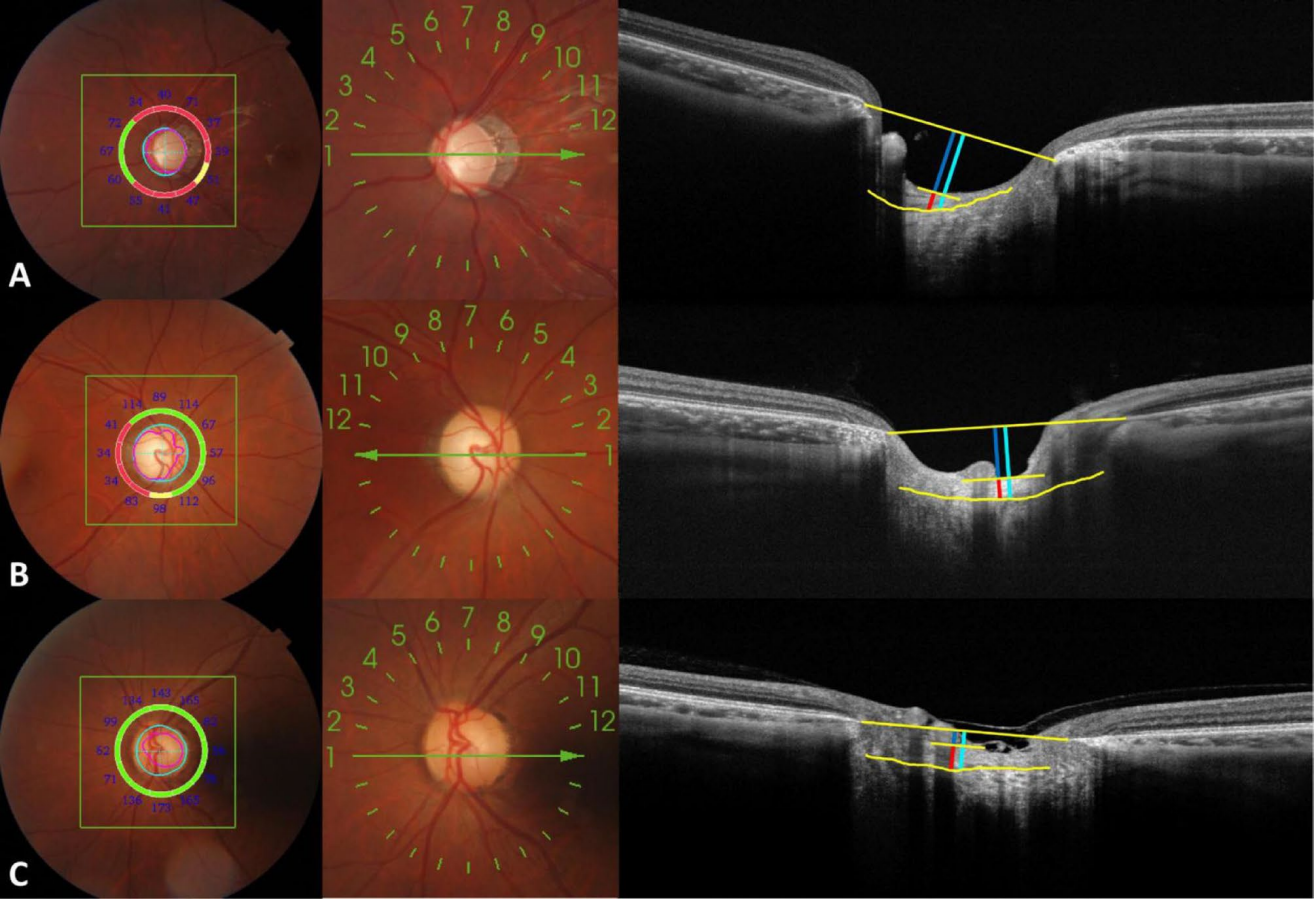
Fundus images and optic disc OCT images in typical cases. (**A**–**C**) showed the fundus and optic disc structure images of a patient with high IOP (group A, IOP = 39.9 mmHg), a patient with moderate IOP (group B, IOP = 24.3 mmHg) and a normal subject (Group C, IOP = 14.3 mmHg). It can be seen that the higher the IOP level, the more serious the defect of optic nerve channel around the optic disc, the higher the C / D ratio, the deeper the CD and LCD on the OCT image, In addition, the curvature of lamina cribrosa increases only for the rough observation on the image. (long yellow line: BMO line on both sides; yellow curve: front surface of sieve plate; dark blue line: CD; light blue line: LCD; red line: PTT).

**Figure 5:**
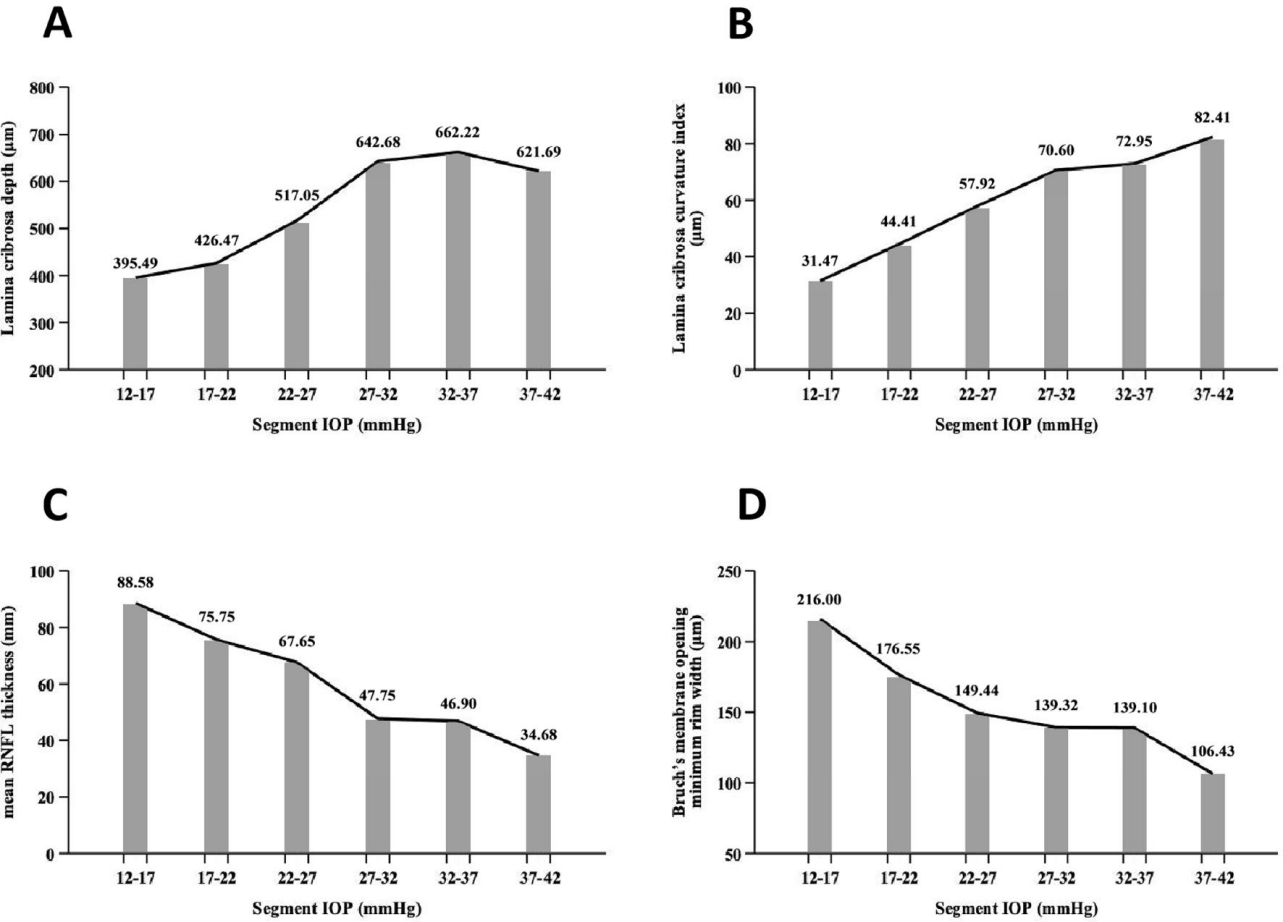
Variation trend of lamina cribrosa parameters under different IOP segments (**A**–**D**) showed the change trend of LCD, LCCI, mRNFLT and BMO-MRW under different IOP segments. It can be seen that LCD gradually presents a gentle development trend with the increase of IOP level, while LCCI will still increase with the increase of IOP. At the same time, according to the change trend of mRNFLT and BMO-MRW, the corresponding relationship between LCCI and these two indicators reflecting the changes of optic nerve layer seems to be more representative than LCD.

Figure 5 illustrates the changes in the optic disc and LC morphology in three typical cases of IOP. As shown in the illustration, the mean IOP of this subject in the first row (group A) was 39.9 mmHg, and the LCD, PTT, and LCCI values were 682.62 μm, 77.95 μm, and 98.24 μm, respectively (Fig. 4A). The subject in the second row (group B) had a mean IOP of 24.3 mmHg, and the LCD, PTT, and LCCI values are 559.12 μm, 203.64 μm, and 64.71 μm, respectively (Fig. 4B). The mean IOP of subjects in the third row (Group C) was 14.3 mmHg, and the values of LCD, PTT, and LCCI were 276.14 μm, 258.81 μm and 23.27 μm, respectively (Fig. 4C).

## Discussion

Through this study, we observed that higher IOP was significantly correlated with the clinical symptoms (BVCA, VF) and increases in CD, LCD, PLCSD, LCCI, and aLCCI, and was also closely related to increases in of PTT and BMO-MRW. Based on the conclusion that the IOP level is the main cause of POAG^36^, the IOP level has a great impact on LC parameters. The higher the IOP level was, the more severe the LC recession and the LC backward bowing.

The clinical symptoms and progress of POAG were mainly manifested in the decline of visual acuity and VF defect. The results of this study showed that the higher the level of IOP is associated, the more serious is the clinical manifestations of POAG, which suggested that a certain relationship is present between different levels of IOP and the progress rate of POAG. LC parameters such as CD, LCD, and PLCSD, were used to reflect the depth level. In previous studies, several articles have pointed out that there is a correlation between the depth of CD and LCD and the progression of glaucomatous VF, while PLCSD is rarely involved due to measurement accuracy^22,37^. The results of this study suggest that the higher the IOP is, the deeper the CD, LCD, and PLCSD. In a linear regression analysis, CD, LCD, and PLCSD deepened with increasing IOP. Therefore, the IOP exerted on the back of the eyeball causes the LC to move backward. However, due to limited measurement accuracy and lack of relevant research support, further studies are needed to confirm the relationship between PLCSD and IOP.

It was also found that the PTT was thinner with increasing IOP, and there was a direct linear correlation between the two factors. The magnitude of the PTT reflects the condition of the prelaminar RNFL. Therefore, PTT will become thinner with increasing IOP, and the severity of glaucoma will also increase. In addition, we also discovered that the LCT of each group exhibited a thinning trend with the increasing IOP; however, there was no significant correlation between them in either the group analysis or the linear regression analysis. Some studies have pointed out that this relationship does exist^24,38^, but some articles have also reported that the LCT of NTG patients exhibited a thinning trend^23^. Therefore, limited by the instruments and research methods of this study, this relationship is worthy of further discussion.

In different groups, LCCI and aLCCI were all associated with IOP, and linear regression analysis revealed that LCCI and aLCCI increased correspondingly with the rise in IOP. LCCI is a relatively accurate indicator of LC deformation because the inclusion of choroidal thickness can be avoided by this method in the determined LCD value, thus avoiding the deviation of LC morphological evaluation^39^. In addition, there are two lines of evidence to demonstrate why the LCCI can more accurately reflect the deformation degree of LC: the influence of the LCCI on choroidal thickness is robust, and the posterior curvature of the LC is evaluated from the insertion point. The newly introduced parameter aLCCI can also reflect the steepness of the LC curve^21^. However, the results showed that the response of aLCCI to LC deformation and RGC axonal stress was not any different from that of LCCI.

Reis et al^40^ and Chauhan et al^41^ proposed a new anatomic parameter, known as BMO-MRW to describe the neuroretinal rim, which is composed of the minimum distance between BMO and the internal limiting membrane (ILM). It has been reported that the RNFL thickness in patients with different stages of glaucoma and the SD-OCT measurement of BMO-MRW were closely associated with VF sensitivity in the 24-2 VF test^42^. In this study, we also used this parameter to analyze its correlation with IOP. The results showed that the BMO-MRW decreased with increasing IOP among the three groups. A significant difference was observed between groups A and C. Linear regression analysis also found that the BMO-MRW gradually narrowed with increasing IOP. However, there was no statistical significance between the BMO-MRW and IOP in the two adjacent groups. The reason may be due to important limitations of the one-dimensional-parameter (i.e., BMO-MRW) shape measurement based on BMO. Especially in a larger optic disc, BMO-MRW was physiologically thinner^43,44^. Meanwhile, the small amount of data and the excessive influence of extreme data may also be one of the reasons for nonconnection.

After grouping IOP with 5 mmHg as a segment and considering the average value of each group for line graph analysis (Fig. 5), we discovered that as the IOP gradually increased, the progression of LCD slowed down when the IOP reached to 35 mmHg, and even stopped deepening or index rebound. Although the rebound of the index may be caused by excess error due to the small amount of data, the decreasing trend was evident, but was not reflected in the LCCI. Therefore, we conclude that there is a stress limit for the sieve plate with increasing IOP. Park et al. found that LCD was the deepest in mild or moderate glaucoma, while there was no significant difference between eyes with mild or moderate glaucoma and eyes with severe glaucoma^37^. The LC is similar to an elastic material^45^, but when the IOP reaches a certain level, the LC gradually reaches the limit, and the progression of CD, LCD, and PLCSD slows down. However, the progression of LCCI may be attributable to the overall level being balanced, the insertion of the LC edge becoming stable, and no fixed substance being present in the center, which leads to the deepening of the central area, consequently stopping the progress of ALID. In addition, the progression of LCD will slow down, and the progression of LCCI will remain unchanged.

The Pearson correlation analysis of the two parameters (LCD and LCCI) and RNFL-related parameters (mean RNFL thickness, BMO-MRW) demonstrated that the correlation level of LCCI may higher compared with that of LCD in high IOP level. In the high IOP level segment, although the two parameters may not show any relationship with RNFL-related parameters due to the lack of data, LCCI still indicated a closer correlation compared with LCD. Therefore, it was concluded that LCCI may be a key indicator in the evaluation of the development of clinical glaucoma because it is more closely associated with the progression of RNFL. Previous studies on experimental early glaucoma monkeys have shown that the LC will not only deform after the onset of glaucoma, but will also reshape due to changes in its biomechanical environment^46,47^. At the same time, a recent study confirmed that eyes with significantly faster VF progression show greater deformation of the LC in the early stage^21^. Deformation will cause strong shear stress on the optic nerve passing through the LC, which will lead to the interruption of the axonal flow of the optic nerve, thus leading VF defects in the corresponding region. The higher the IOP, the more serious the LC deformation, which is proportional to the degree of optic nerve necrosis. Therefore, the role of LCCI should not be ignored in assessing the severity of glaucoma.

In this study, there are also some limitations, and the findings must be explained based on the limitations. First, the mean deviation (MD) in the VF test was not used as a clinical indicator because it was not widely examined in all subjects who underwent this test, which resulted in a small number of subjects with MD and did not meet the requirements for sample size in this study. However, we have reasons to believe that MD, as a parameter measuring the relationship between changes in LC parameters and clinical manifestations, will make the results more accurate. We hope that follow-up research can be further carried out for MD. Second, the clarity limitation of OCT may cause errors in the measurement of deeper structures (LCT and PLCSD). Therefore, to navigate this problem as much as possible, we only selected the two-point clear images of transverse scanning and vertical scanning around the optic disc by the radial scanning mode. Although the results are consistent with the findings from most articles, the average parameters obtained from the analysis of all 12 o’clock images are bound to have more positive findings. At the same time, OCT parameters were measured subjectively by clinicians, so there will be inevitable bias in measurement value. Secondly, we did not adjust the amplification effect according to the individual differences in AL. Littman believes that, as the AL increases, the uncorrected transverse measurements decrease, which may affect the results of LC measurements^48^. However, most of the AL was distributed across a relatively narrow range (22.23–26.70 mm). Therefore, we carefully evaluated whether the difference in AL would not produce a critical impact on our results. Additionally, the influence of AL difference on the measurement results of each LC parameter needs to be studied in the future. Thirdly, since the fluctuation of IOP had uncertainty, we had excluded the subjects with unstable IOP as much as possible. Yet, it was still not guaranteed that the patients were continuously affected by constant IOP. Therefore, the measurement of mean IOP could not fully represent the influence of IOP on the development of POAG. Sommer et al*.* pointed out that in addition to IOP, the factors affecting glaucoma may also be influenced by age, family history, blood pressure, optic disc structure and other factors. Therefore, other unrelated variables will also interfere with the research results to a certain extent, as this study is a cross-sectional study^49^. Therefore, a follow-up cohort study of control variables may be able to further verify these results. Given the uncertain causal relationship between RNFL change and LC shift, the course of some POAG cases is characterized by long-term and chronic development, and sometimes obvious LC changes may not be observed in short-term (within one year) follow-ups. Therefore, further longitudinal studies are warranted to confirm it. And other biases such as positive correlation of binocular subjects and selection bias will have different effects on the research results.

In conclusion, different IOP levels have an impact on clinical symptoms (VF, BCVA) and LC parameters. A higher IOP leads to worse VF and visual acuity, the backward bowing of the LC, deformation of the LC, and thinning of the RNFL around LC. Moreover, LCCI may be a better indicator of the severity of glaucoma than LCD. Therefore, it is suggested that the immediate control of high IOP in clinical practice will minimize the severity of glaucoma. The effect of IOP on glaucoma may be triggered by serious morphological changes in LC, which suggests that the assessment of LC morphology with OCT imaging may be more suitable to monitor the progression of glaucoma in the follow-up and early diagnosis. In the high IOP level, LCCI can evaluate glaucoma better. This may be due to the change of LCCI as the key indicator of RNFL change. In future studies, we hope to dynamically observe the effect of IOP on LC parameters and further investigate the correlation between LCCI and the progression of glaucoma.

### Ethical approval and consent to participate

All procedures performed in studies involving human participants were in accordance with the ethical standards of the institutional and/or national research committee and with the 1964 Helsinki declaration and its later amendments or comparable ethical standards. This study obtained the ethical clearance from the Beijing Tongren Hospital Ethical Committee, according to the Helsinki Declaration. Written informed consent was obtained from all participants. For those who were illiterate or blindness, we read the consent form to them and asked them to mark the consent form with an inked forefinger, and the consent form with an inked forefinger that obtained from illiterate participant was also approved by the Ethics committee.

## Data Availability

The data of the current study are available from the corresponding author on reasonable request.

## Abbreviations

aLCCI: Adjusted LCCI
ALID: Anterior laminar insertion depth
AL: Axial length
BMO: Bruch’s membrane opening
BMO-MRW: Bruch’s membrane opening minimum rim width
CD: Cup depth
CV: Cup volume
CDR: Cup/disk ratio
EDI-OCT: Enhanced depth imaging-OCT
ILM: Internal limiting membrane
IOP: Intraocular pressure
LC: Lamina cribrosa
LCCI: Lamina cribrosa curvature index
LCD: Lamina cribrosa depth
LCT: Lamina cribrosa thickness
mALID: Mean ALID
MD: Mean deviation
ONH: Optic nerve head
PLCSD: Posterior lamina cribrosa surface depth
PTT: Prelaminar tissue thickness
RGC: Retinal ganglion cell
RNFL: Retinal nerve fiber layer
RNFL thickness: Retinal nerve fiber layer thickness
VF: Visual field
